# Baveno VII Criteria Is an Accurate Risk Stratification Tool to Predict High-Risk Varices Requiring Intervention and Hepatic Events in Patients with Advanced Hepatocellular Carcinoma

**DOI:** 10.3390/cancers15092480

**Published:** 2023-04-26

**Authors:** Claudia Wing-Kwan Wu, Rashid Nok-Shun Lui, Vincent Wai-Sun Wong, Tsz-Fai Yam, Terry Cheuk-Fung Yip, Ken Liu, Jimmy Che-To Lai, Yee-Kit Tse, Tony Shu-Kam Mok, Henry Lik-Yuen Chan, Kelvin Kwok-Chai Ng, Grace Lai-Hung Wong, Stephen Lam Chan

**Affiliations:** 1 Medical Data Analytics Centre, Hong Kong SAR, China; 2 Department of Medicine and Therapeutics, Prince of Wales Hospital, Hong Kong SAR, China; 3 Institute of Digestive Disease, Prince of Wales Hospital, Hong Kong SAR, China; 4 AW Morrow Gastroenterology and Liver Centre, Royal Prince Alfred Hospital, Camperdown, NSW 2050, Australia; 5 State Key Laboratory of Translational Oncology, Department of Clinical Oncology, The Hong Kong Cancer Institute, Hong Kong SAR, China; 6 Union Hospital, Hong Kong SAR, China; 7 Department of Surgery, The Chinese University of Hong Kong, Hong Kong SAR, China

**Keywords:** Baveno criteria, hepatocellular carcinoma, systemic therapies, high risk varices, liver stiffness measurement, platelet

## Abstract

**Simple Summary:**

Patient with hepatocellular carcinoma are at an increased risk of variceal haemorrhage, and often require endoscopic workup to look for the presence of high-risk varices prior to starting systemic therapy. The Baveno VII criteria has been a well-validated non-invasive risk stratification tool in predicting high-risk varices for patients with liver cirrhosis, but data on its use in the hepatocellular carcinoma population is lacking. In this study, we successfully validated the Baveno VII criteria as a safe and accurate modality in predicting high-risk varices and hepatic events in patients with hepatocellular carcinoma.

**Abstract:**

The Baveno VII criteria are used in patients with liver cirrhosis to predict high-risk varices in patients with liver cirrhosis. Yet its use in patients with advanced hepatocellular carcinoma (HCC) has not been validated. HCC alone is accompanied with a higher variceal bleeding risk due to its association with liver cirrhosis and portal vein thrombosis. The use of systemic therapy in advanced HCC has been thought to further augment this risk. Upper endoscopy is commonly used to evaluate for the presence of varices before initiation of treatment with systemic therapy. Yet it is associated with procedural risks, waiting time and limited availability in some localities which may delay the commencement of systemic therapy. Our study successfully validated the Baveno VI criteria with a 3.5% varices needing treatment (VNT) missed rate, also with acceptable <5% VNT missed rates when considering alternative liver stiffness (LSM) and platelet cut-offs. The Baveno VII clinically significant portal hypertension rule-out criteria (LSM < 15 kPa and platelet >150 × 10^9^/L) also revealed a low frequency (2%) of hepatic events, whilst the rule-in criteria (LSM > 25 kPa) was predictive of a higher proportion of hepatic events (14%). Therefore, our study has successfully validated the Baveno VII criteria as a non-invasive stratification of the risk of variceal bleeding and hepatic decompensation in the HCC population.

## 1. Introduction

Variceal bleeding is a dreaded complication of portal hypertension, which commonly arises in diseased states such as liver cirrhosis or advanced hepatocellular carcinoma (HCC), and is associated with significant morbidity and mortality [1,2,3]. In patients with cirrhosis, upper gastrointestinal endoscopies are usually performed to screen for varices and guide prophylaxis strategies. For some asymptomatic patients with advanced HCC, the decision on whether to proceed with endoscopy is made on a case-by-case basis.

With the advent of systemic therapy for the treatment of HCC, such as multikinase inhibitors sorafenib [4] and lenvatinib [5] that exhibit vascular endothelial growth factor inhibition (VEGFi) properties, the concern for their association with increased bleeding risk has led to increased demand for variceal screening by endoscopy prior to treatment initiation. A prospective study published in 2016 assessing the gastrointestinal bleeding risk of sorafenib in the treatment of advanced HCC showed that there was an 8% risk of gastrointestinal bleed during sorafenib treatment, all of which were secondary to variceal bleeding [6]. The study stressed the need for screening oesophagogastroduodenoscopy (OGD) to look for oesophageal varices (OV) prior to starting sorafenib and suggested the importance of achieving good OV obliteration before treatment initiation [7]. However, endoscopy is an invasive procedure that comes with risks and may not be preferred especially in patients who may already be frail from an advanced malignancy [8,9]. With the ongoing COVID-19 pandemic, many elective procedures may have been canceled or delayed, which may lead to further postponement in treatment initiation. At present, only a limited number of studies have examined the OV bleeding risk of advanced HCC patients on systemic therapy, and there is currently no clear international consensus on the threshold for screening OGD prior to treatment initiation [7,10].

The Baveno VI criteria with a liver stiffness measurement (LSM) of ≤20 kPa by transient elastography (TE) and a platelet count (PLT) of ≥150 × 10^9^/L has long been validated as a safe non-invasive tool predictive of a low risk of clinically significant varices, negating the need for invasive endoscopic investigation for OV. The updated Baveno VII consensus extends the application to the diagnosis of compensated advanced chronic liver disease (cACLD) and clinically significant portal hypertension (CSPH), which predicts the risk of developing decompensated liver disease and variceal bleeding. The Baveno VII criteria recommends that patients with an LSM ≤ 15 kPa with PLT > 150 × 10^9^/L has a >90% predictive value in ruling out CSPH, which translates clinically into a low probability of high-risk varices, thus obviating the need for screening endoscopy until yearly follow-up LSM increases to ≥ 20 kPa and/or PLT ≤150 × 10^9^/L. It also recommends that an LSM >25 kPa can sufficiently diagnose CSPH [11].

Whilst the Baveno VII consensus is applicable to all patients with liver cirrhosis, its use in advanced HCC patients has not been well defined. HCC has often been studied as an endpoint in non-invasive assessment of liver cirrhosis, with LSM used as a predictor for HCC risk. Studies have shown that a high LSM is associated with an increased risk of HCC development. However, the use of LSM in defining the risk of varices needing treatment (VNT) in the HCC population has not been studied. It is often thought that liver lesions such as HCC or malignant vascular invasions can alter results of TE by under- or over-estimating LSM. Measurements can inadvertently be performed on the tumour mass instead of the liver itself and provide us with an inaccurate LSM that does not reflect prognosis. Studies of LSM in HCC patients have previously provided conflicting results [12,13]. Our study aims to investigate both the Baveno VI criteria as a non-invasive risk stratification tool in assessing the presence of VNT, as well as the extended Baveno VII criteria in predicting CSPH in patients with advanced HCC with or without systemic therapy.

## 2. Materials and Methods

### 2.1. Study Design Overview and Subjects

This was a post hoc analysis of a prospectively recruited cohort study of patients with a confirmed diagnosis of HCC referred to the multidisciplinary Joint Hepatoma Clinic at the Prince of Wales Hospital, Hong Kong Special Administrative Region, China, from March 2013 to April 2019. Patients underwent TE examination before HCC treatment, and received at least one OGD examination at least 6 months prior to or up to 1 month after systemic therapy initiation, allowing for the potential waiting time needed for an OGD appointment. Key inclusion criteria included patients with confirmed diagnosis of HCC according to the European Association for the Study of the Liver (EASL) Clinical Practice Guidelines [14], and were able to provide written informed consent. We included patients of Barcelona Clinic Liver Cancer (BCLC) staging A–C, who were under consideration for receiving systemic therapy. For comparison, we also included patients who were offered therapies such as hepatectomy, embolization therapy or best supportive care. Key exclusion criteria included patients with Eastern Cooperative Oncology Group (ECOG) performance status of 3 or higher; BCLC stage D, presence of poorly controlled ascites; and life expectancy of 8 weeks or shorter. The study was approved by the Joint Chinese University of Hong Kong–New Territories East Cluster Research Ethics Committee (CRE Ref. No. 2011.444).

### 2.2. Clinical and Laboratory Evaluation

Baseline evaluation included a full medical history, physical examination, anthropometric measurement and blood tests, which were collected from each patient via the electronic medical record system during enrolment prior to the performance of TE. Additional data were also retrospectively collected from the electronic medical record system and analysed according to the latest Baveno VII criteria. Relevant virologic assays were performed according to their aetiology. Viral hepatitis B and C infections were diagnosed by positive serology tests for serum hepatitis B surface antigen (HBsAg) and hepatitis C virus antibodies (anti-HCV), respectively. Non-alcoholic fatty liver disease was diagnosed by ultrasonography and histology after exclusion of excessive alcohol consumption and other secondary causes of fatty liver [15].

### 2.3. Transient Elastography Examination

Details of the technical background and examination procedure of LSM have been described elsewhere [16]. LSM were performed using transient elastography machine (FibroScan, Echosens, France). LSM, expressed in kPa, is considered reliable only if 10 successful acquisitions were obtained and the ratio of interquartile range over the median (interquartile range (IQR)/LSM) is ≤0.3 [17]. Patients with unreliable LSM results would have the examination repeated immediately; the results would not be analysed if these remained unreliable upon repeated examination. One experienced operator (GLHW) performed all the TE assessments. She had more than five years of experience and had undergone more than 1000 TE examinations before this study started. The interquartile range (IQR), IQR range/LSM ratio and success rate of LSM at all visits were 25.4 (11.9–66.8) kPa, 15.2 (7.3–22.6)% and 100 (83–100)%, respectively. The operator was blinded to all clinical data and the diagnoses of the patients. All TE examinations were conducted before patients were initiated on any systemic therapy.

### 2.4. Upper Endoscopy

Upper endoscopic examination was performed by well-trained endoscopists who had performed at least 100 upper endoscopic examinations. We have previously reported that the inter-observer agreement ranged from 0.88 to 0.93 for any varices and 0.95 to 1.0 for clinically significant varices for these endoscopists [18]. Both endoscopists and patients were blinded to the LSM results at the time of endoscopic examination. The endoscopic findings were recorded in a standard format with relevant oesophageal and gastric varices findings recorded and graded according to international guidelines [19]. All patients had screening upper endoscopies conducted between 6 months prior to up to 1 month after initiation of any systemic therapy, to allow for any delay in endoscopy booking appointment.

### 2.5. Systemic Therapies for HCC

Enrolled patients were offered a choice of standard systemic treatment according to the latest guidelines, which were locally available at the time of assessment. During the study period from 2013 to 2019, sorafenib (since 2013) or lenvatinib (since 2018) were offered to patients as the first-line therapy while options for second-line therapy include regorafenib (since 2017), pembrolizumab and nivolumab (since 2018). Those who did not receive systemic therapies may have been offered alternative therapies such as surgical intervention, transarterial chemoembolization (TACE), etc. Alternatively, they may have opted for palliative care.

### 2.6. Clinical Assessments during Follow-Up

After TE examination, patients were followed up once every 3 to 6 months. During each visit, patients’ symptoms, clinical events, drug history and adherence were recorded. Haemoglobin, clotting profile, renal and liver biochemistries and alpha-fetoprotein were checked at every visit. Computed tomography or ultrasonography of the abdomen was performed every 3 to 6 months to monitor treatment response [14]. All hospitalisation and endoscopy records were captured.

### 2.7. Clinical Outcomes

The primary outcome was the cumulative incidence rate of VNT, defined as all grade II to III varices (≥5 mm), small varices in patients with Child–Pugh C cirrhosis and/or varices with stigmata of recent haemorrhage such as red-wale signs, fibrin clot or active bleeding. No pharmacological or endoscopic treatment was offered to patients with no varices, while non-selective beta-blockers (NSBB) such as propranolol or carvedilol were given to patients with VNT. Endoscopic banding ligation therapy was performed in patients with actively bleeding oesophageal varices, varices of any size identified in the presence of blood in the oesophagus and/or stomach or varices of any size with the presence of red-wale signs and/or fibrin plugs, or in case of contraindications or intolerance to propranolol and carvedilol [19]. Standard endoscopic banding ligation therapy was performed with a multi-band ligation device. Ligation was confined to the distal oesophagus starting at the gastroesophageal junction, ligating first the most prominent varix or the bleeding varix in the case of active bleeding. Other varices were ligated sequentially in a cephalad direction. Patients with bleeding gastric varices were treated with histoacryl glue injection per departmental protocol.

The secondary outcome was a composite endpoint of the cumulative incidence rates of hepatic events excluding HCC that occurred during the follow-up period, including variceal bleeding, ascites, spontaneous bacterial peritonitis (SBP), hepatic encephalopathy, hepatorenal syndrome (HRS) and liver-related death. Ascites was defined as free peritoneal fluid identified on ultrasound or computer tomography scans, or as clinically evident ascites confirmed by paracentesis. SBP was defined as an ascitic fluid polymorph count of 250/mm^3^ or above with or without positive bacterial culture.

### 2.8. Statistical Analysis

Data were analysed using Statistical Product and Service Solutions (SPSS) version 27.0 (SPSS, Inc., Chicago, IL, United States), and R software (4.0.5; R Foundation for Statistical Computing, Vienna, Austria). Continuous variables were expressed in mean ± standard deviation or median (IQR), as appropriate, while categorical variables were presented as frequency (percentage). Differences in proportion of patients who developed VNT between groups defined by different cut-offs of LSM and PLT were analysed by chi-square test. A ≤5% VNT missed rate is considered acceptable. The Kaplan–Meier method was used to estimate the cumulative incidence of VNT and hepatic events with a 95% confidence interval (CI); a log-rank test was used to compare the cumulative incidence of different risk groups. Predictors of VNT and hepatic events were assessed using univariable and multivariable competing risk Fine–Gray regression model. In analysis of VNT and hepatic events, the Fine–Gray competing risk regression model was also applied with death as a competing risk. All statistical tests were two-sided with *p* < 0.05 being taken as statistically significant.

## 3. Results

### 3.1. Patient Characteristics

Within a cohort of 225 patients from March 2013 to April 2019, a total of 200 patients were included in this study after exclusion of patients with poor ECOG status or those with refractory ascites. The baseline characteristics of the patients, including the demographics, organ function as well as tumour stages and characteristics, are summarised in Table 1. The median age was 61 years with 70% having chronic hepatitis B infection and 7.5% having chronic hepatitis C infection. A number of 125 (63%) and 159 (80%) patients had PLT ≥ 150 × 10^9^/L and ≥ 110 × 10^9^/L, respectively. Their median (IQR) Child–Turcotte–Pugh (CTP) score was 6 (5–7); 135 (68%) and 64 (32%) patients had CTP grade A and B, respectively. Only one patient had CTP grade C. The majority of patients (n = 124, 62%) had HCC of BCLC stage C, whereas 25 (13%) patients had stage A and 51 (26%) patients had stage B. The median follow-up duration was 11.9 months. A total of 76 patients received systemic therapy, with 69 receiving sorafenib and 4 receiving lenvatinib. Five patients received nivolumab, two of them as second-line therapy after sorafenib and one of them as second-line therapy after lenvatinib. One patient received pembrolizumab. All systemic therapies were started after TE was completed. A total of 27 patients had prior therapy before receiving systemic therapy. A number of 8 of them had prior partial hepatectomy, 17 received prior TACE and 2 received prior radiofrequency (RF) ablation therapy (Supplementary Table S1). There was no clinically significant difference in the basic demographics of patients given systemic therapy when compared with those who were not on systemic therapy.

There were 124 patients who did not receive systemic therapy. Of these patients, 75 patients received alternative therapy, whilst 49 patients received no treatment or opted for palliative care. Within the patients who received alternative therapy, 49 received TACE, 20 received partial hepatectomy and 6 received RF ablation (Supplementary Table S2).

### 3.2. Liver Stiffness Measurement

The results of LSM are summarised in Table 1. The median LSM for the whole study population was 25.4 kPa (Range: 11.9–66.8 kPa). 60 (30%) patients had LSM ≤ 15 kPa, 13 (6.5%) patients had LSM 15–20 kPa, 15 (7.5%) patients had LSM 20–25 kPa and 88 (44%) patients had LSM >25 kPa. A number of 24 patients defaulted their TE measurements and therefore do not have LSM data. A relatively higher proportion of patients who were not on systemic therapy had LSM ≤ 20 kPa compared with those on systemic therapy (42% vs. 27%; *p*-value: 0.059). Right-sided tumour involvement was present in 91 (45.5%) patients. The median LSM was 17.2 (10–46.7) in those with right-sided involvement, and 35.8 (14.1–75) in those without right-sided involvement (*p*-value: <0.001).

### 3.3. Baveno Criteria and Other Combinations of LSM and PLT

The proportion of patients that fulfilled the Baveno VI criteria, i.e., LSM ≤ 20 kPa and PLT > 150 × 10^9^/L, was 20.5% (41 patients) in total; amongst these, 13 patients (32% in the group) received systemic therapy, and 28 patients (68%) did not receive systemic therapy.

Whilst exploring alternative cut-offs of LSM and PLT, it was found that a lower LSM cut-off ≤ 15 kPa with the same PLT > 150 × 10^9^/L included fewer patients (18.5%). A lower cut-off of PLT > 110 × 10^9^/L resulted in more patients being included, with 27% having LSM ≤ 20 kPa with PLT > 110 × 10^9^/L, and 23.5% having LSM ≤ 15 kPa with PLT > 110 × 10^9^/L. On the other hand, with a higher LSM cut-off of ≤ 25 kPa, more patients would have fulfilled the combined LSM-PLT criteria (26.5% for PLT > 150 × 10^9^/L, and 33.5% for PLT > 110 × 10^9^/L), resulting in more patients being spared from screening endoscopy.

### 3.4. Varices in Patients Who Did or Did Not Receive Systemic Therapies

A total of 50 (25%) patients had any varices diagnosed by OGD; by far the majority of patients had oesophageal varices (48 patients, 24%), whereas only 4 (2%) patients had gastric varices (Table 2). Further, 2 (1%) of them had both oesophageal and gastric varices. Within both groups who received systemic therapy and the group who did not, OV was present in 24% of patients. GV was present in 2.6% of patients who received systemic therapy, and in 1.6% of patients who did not receive systemic therapy. Over a median (IQR) follow-up duration of 11.9 (4.6–39.6) months, acute variceal bleed (AVB) occurred in 11 (5.5%) patients, with 8 (3.5%) patients having bleeding OV and 3 patients (1.5%) with bleeding gastric varices. The aetiologies of the AVB were found to be related to disease progression in 6 patients and portal vein thrombosis (PVT) in 5 patients. All 3 AVB events in patients who received systemic therapy occurred in patients who received sorafenib after the onset of starting treatment. Of these, 2 of them had pre-treatment OGD completed with grade 1 OVs detected. Sorafenib was given for only 2 months for both of these patients and was stopped due to poor patient tolerance. These AVB events occurred at least 4 months after cessation of systemic therapy in both cases. The other patient developed bleeding GV 4 months after sorafenib initiation. Sorafenib treatment was eventually stopped due to disease progression. All 3 patients with AVB did not meet the Baveno VI criteria and thus would have required a pre-treatment OGD anyway and should not be considered a failure of the Baveno criteria.

Out of the 8 patients who developed AVB and did not receive systemic therapy, only 1 fulfilled the Baveno criteria. However, it should be noted that the bleeding event occurred 3 years after the index TE. The patient had since then experienced disease progression and developed PVT, which would have likely increased his portal pressure and therefore risk of variceal haemorrhage.

Most patients with VNT had CTP grade A cirrhosis (12%). A higher proportion of patients with VNT had BCLC stage C disease (21 patients; 11%), as compared to 8 patients (4%) who had BCLC stage A disease. A higher BCLC staging was associated with a significantly higher VNT event rate (*p*-value: 0.04; Supplementary Figure S1). A number of 12 out of 75 patients (16%) with PVT had VNT; 33 out of 125 patients (26%) without PVT had VNT. The presence of PVT did not significantly impact the rate of clinically significant varices in this cohort (*p*-value: 0.088). Bleeding events occurred in 5.3% patients with PVT and 4.8% patients without PVT.

### 3.5. VNT and Baveno VII Criteria

**Figure 1:**
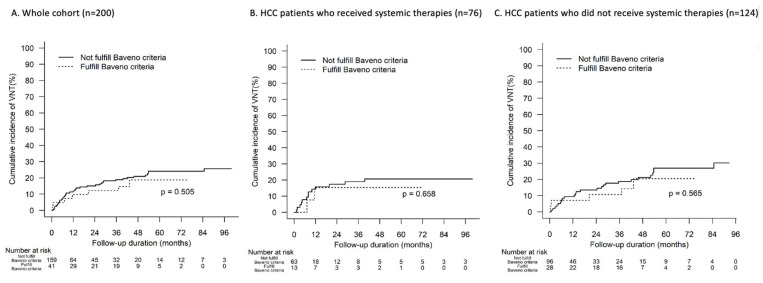
Time to VNT according to Baveno VI criteria.

VNT occurred in 45 (23%) patients, accounting for 20% of patients who received systemic therapy and 24% of patients who did not receive systemic therapy. Only 7 (3.5%) patients with VNT would be missed by the Baveno VI criteria, whereas 38 patients (19%) would have been correctly identified as not fulfilling the Baveno VI criteria (*p*-value: 0.5; Figure 1). Use of systemic therapy did not result in a significantly different rate of VNT or bleeding varices in our cohort (*p*-value: 0.44; Supplementary Figures S2 and S3).

A number of 2 patients (2.6%) who received systemic therapy and 5 patients (4%) who did not receive systemic therapy had VNT that were missed by the Baveno criteria. When analysing VNT missed rates according to whether or not patients received systemic therapy, it was noted that a higher (although insignificant) percentage of VNT missed rates were present in patients who did not receive systemic therapy (Table 2).

In exploring alternative LSM and PLT cut-offs, most remained valid in predicting a low rate of VNT, except for the most lenient cut-off of LSM ≤ 25 kPa and PLT > 110 × 10^9^/L where the VNT missed rate exceeded 5%. Using the alternate LSM ≤ 20 kPa and PLT > 110 × 10^9^/L cut-off, which includes at least one-fourth of patients (27%), the VNT missed rate just approaches the 5% threshold. In those who did not receive systemic therapy, there was a >5% VNT missed rate when applying more lenient cut-offs, such as LSM ≤ 20 kPa and PLT > 110 × 10^9^/L, or LSM ≤ 25 kPa and PLT > 110 × 10^9^/L (Table 2).

### 3.6. Hepatic Events and Baveno VII Criteria

Apart from liver-related deaths, a total of 56 patients developed any hepatic events (28%) that occurred within the study follow-up period, with 24% of them who received systemic therapy and 31% who did not. These included ascites (44 patients, 22%), hepatic encephalopathy (17 patients, 8.5%), AVB (11 patients, 5.5%), hepatorenal syndrome (6 patients, 3%) and SBP (4 patients, 2%) (Table 3). Liver-related deaths, including death from HCC or variceal bleeding, occurred in 128 patients (64%) during the follow-up period. Only one patient had a prior episode of hepatic decompensation before baseline FibroScan measurement; excluding that patient did not affect the main study findings.

**Figure 2:**
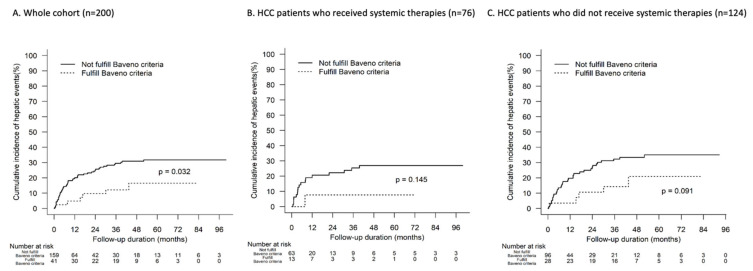
Time to hepatic events according to Baveno criteria.

Hepatic events occurred in 15% (6 out of 41) patients who fulfilled the Baveno VI criteria (LSM ≤ 20 kPa and PLT > 150 × 10^9^/L), compared with 31% (50 out of 159) patients who did not. Hepatic events occurred in 4 (2%) patients who fulfilled the extended Baveno VII criteria of LSM <15 and PLT >150 to rule out CSPH, whereas 28 (14%) patients had LSM >25 kPa, which rules in the diagnosis of CSPH. Those who fulfilled the Baveno criteria were significantly less likely to develop hepatic events compared with those who did not fulfil the criteria (*p*-value: 0.032; Figure 2A).

Of those who fulfilled the Baveno VI criteria, only 1 patient who received systemic therapy developed hepatic events, whilst 5 of 28 patients (18%) who did not receive systemic therapy developed hepatic events. In the subgroup analyses by systemic therapy use, the difference in cumulative incidence of hepatic events in patients who fulfilled the Baveno criteria versus those who did not becomes insignificant (*p*-value: 0.145 and 0.091; Figure 2B,C).

Liver-related deaths occurred in 128 (64%) patients within the entire cohort. It occurred in 16 out of 41 patients (39%) who fulfilled the Baveno VI criteria, with HCC being the primary cause of death for all cases. During the post hoc analysis, 112 out of 159 patients (70%) who did not fulfil the Baveno criteria had liver-related deaths, with HCC being the main cause of death except for 1 patient who died of OV bleeding. A total of 15 out of 37 patients (40%) with CSPH rule-out criteria had liver-related deaths, whilst a much higher proportion of patients (67 out of 88 patients; 76%) who fulfilled the CSPH rule-in criteria had liver-related deaths. As well, 1 patient had OV identified on index OGD prior to sorafenib initiation. Sorafenib therapy was given for 3 months then stopped due to poor liver function. The patient then succumbed 2 months after treatment cessation with an episode of OV bleeding, despite endoscopic variceal ligation.

When looking at alternative LSM and PLT cut-offs, the overall number of hepatic events apart from liver-related deaths was low (4–12 patients; 2–6%). As expected, a higher LSM and a lower PLT cut-off was associated with a higher number of hepatic events. When considering liver-related deaths, the proportion of hepatic deaths remained below 20% (15–34 patients; 7.5%–17%) at the different LSM and PLT cut-offs. Interestingly, patients who did not receive systemic therapy had a higher percentage of hepatic events (albeit being statistically insignificant) in all subgroups compared with those on systemic therapy (Table 3). Overall, there was a small but insignificant difference in overall survival between the group on vs. not on systemic therapy (7.9 months vs. 15.4 months; 0.067).

### 3.7. Predictors of VNT and Hepatic Events

Both univariate and multivariate analyses were performed to detect variables associated with risk of VNT and hepatic events. Only PLT was found to be an independent predictor of both VNT and hepatic events (*p*-value: 0.001 and 0.005, respectively) according to the multivariate analysis (Table 4 and Table 5). Other factors such as age, gender, BCLC or Child–Pugh staging, use of systemic therapy, presence of PVT and even LSM were not found to be significant in this study.

The Baveno VII criteria have been widely studied since their publication, with many studies validating their use in patients with liver cirrhosis. However, the application of the Baveno VII criteria has never been validated in advanced HCC patients, and it is thought that the presence of hepatic tumours or vascular invasion modifying the liver architecture would significantly affect LSM and PLT counts, and that patients with more advanced HCC may develop more rapidly progressive disease and liver-related complications [20,21]. The accuracy in predicting VNT in HCC is further complicated by the use of systemic therapy. Many guidelines and studies recommend pre-treatment endoscopy evaluation for varices, however little real-world data exist on the OV bleeding risk in this subgroup.

A recent study examining the validity of Baveno VII criteria of HCC patients who underwent hepatectomy has found these criteria to be consistently applicable to screen for VNT across HCC patients of different BCLC stages [22]. To the best of our knowledge, our study is one of the first studies that provides long-term follow-up data to support the validity of the Baveno criteria as an accurate risk stratification tool to screen for VNT in advanced HCC patients, with an acceptable VNT missed rate of <5%. More lenient alternative cut-offs of LSM ≤ 25 kPa and PLT > 150 × 10^9^/L was also found to have an acceptable missed VNT rate of ≤5%, regardless of the use of systemic therapies.

The outcome of this study implies that for a selected number of patients at low risk of developing variceal haemorrhage, the need for a mandatory screening OGD before treatment initiation can be obviated with the application of the Baveno VI criteria. Patients who were on systemic therapy also did not have a significantly higher risk of variceal bleeding compared to those who were not given systemic therapy. This suggests that the overall bleeding risk from systemic therapy use may not be as high as previously thought, and the use of systemic therapy also did not impact the validity of Baveno criteria. 

The advantages of utilizing the Baveno criteria in advanced HCC patients are multi-fold. First, the application of the criteria reduces endoscopy workload and saves healthcare resources. Second, avoiding delay in the waiting time of endoscopic screening allows earlier initiation of HCC therapy and may alter prognosis. Third, its non-invasive nature also minimizes the risk of endoscopy-related complications and morbidity, as OGDs are often poorly tolerated by many patients [23].

Various other non-invasive tools have also been assessed to predict VNT. Many studies have gone into exploring spleen stiffness measurement (SSM), which uses a TE machine with a dedicated spleen stiffness module to measure spleen stiffness as a surrogate marker for portal hypertension and OV risk in patients with viral hepatitis. This is undertaken on the basis that portal hypertension induces splenic congestion, which provokes architectural changes in the splenic vasculature. Combining the liver and spleen stiffness measurement (LSSM) not only resulted in very few missed VNT (sensitivity and negative predictive values as high as 91% and 96%, respectively), it also addresses the critical limitation of a substantial “grey zone” when using Baveno VI criteria alone, resulting in a higher proportion of OGDs spared (50% vs. 33%) [24]. Although SSM better correlates with portal pressure, the accuracy of SSM requires an experienced operator and is associated with a longer learning curve. In addition, SSM has also not been validated in patients with HCC. Thus, we believe that the Baveno VII criteria would serve as a suitable alternative approach that is more accessible, widely applicable and more easily implementable.

Furthermore, our study also validated Baveno VII criteria as a predictor for the risk of hepatic events in advanced HCC patients. As suggested in the Baveno VII consensus, LSM in itself is a surrogate marker for the risk of hepatic events. Significant reduction in LSM by >/ = 20% or to <10 kPa is associated with a reduced risk of decompensation and liver-related death. Incremental increase in LSM is predictive of an increased risk of decompensated hepatic events and liver-related death. This trend was similarly observed in this study. Overall, the event rates for various hepatic events in those who fulfilled the extended Baveno VII criteria was low at 2%, compared to 14% in those who did not fulfil the Baveno VII criteria. Death from HCC occurred in a much lower percentage (40% vs. 76%) of patients who fulfilled the extended Baveno VII criteria compared with those who did not. This implies that the use of Baveno criteria in HCC patients not only predicts VNT, but also infers prognosis.

Patients who were on systemic therapy on the whole had fewer VNT and hepatic events compared with those who were not on any treatment (Table 2 and Table 3). This included a lower percentage of AVB in the systemic therapy group. This may possibly be attributed to the therapeutic effects of systemic therapy; however, we cannot rule out the confounding effects of meticulous patient selection (i.e., excluding those at higher bleeding risk or those with decompensated liver cirrhosis) prior to treatment commencement. The high proportion (40%) of patients who received supportive treatment within those who did not receive systemic therapy may also contribute to this. Curiously, there was a higher proportion of patients without PVT who had VNT compared with those who had PVT (26% vs. 16%; Table 2). This is unexpected as PVT increases the risk of portal hypertension and therefore should increase the chance of VNT development [25]. However this difference was not statistically significant and bleeding events were similar in the two groups (4.8% vs. 5.3%). Within a subgroup analysis, patients with or without PVT both had acceptable VNT missed rates when applying the Baveno VI criteria (1% vs. 2.5%; *p*-value: 1; Supplementary Table S3).

The incidence of hepatic events was significantly lower in those who fulfilled the Baveno criteria when compared with those who did not, although this difference is not significant when analysing according to subgroups of patients with or without systemic therapy. Given that there was only a relatively small number of patients on systemic therapy who fulfilled the Baveno criteria, further analysis with a larger sample size is warranted.

Even if we apply the Baveno criteria, there will still be a significant proportion of upper endoscopies that could have been spared; 78% of our cohort did not develop VNT during our study period, yet the Baveno VII criteria was able to spare 21% of our cohort only. This points towards a low specificity of the Baveno VI criteria. Therefore, exploring alternative cut-offs in LSM and PLT counts can be a useful strategy to further lessen the number of unneeded endoscopic interventions and hopefully improve specificity. In regions where limited resources are available, or a long waiting time for endoscopies are expected, a more lenient cut-off may be beneficial for better resource allocation whilst retaining a low VNT missed rate. However, caution should be exercised in pushing the boundaries beyond LSM ≤ 25 kPa and PLT > 110 × 10^9^/L, as our study demonstrated >5% VNT missed rate with this cut-off.

It is important to note that tumours with right-sided involvement were found to have a significantly lower median LSM than those without right-sided involvement. This points towards LSM potentially being underestimated in those with right-sided tumour involvement. Consistent with previous studies, the presence of tumour-related factors (such as tumour size, number, vascular invasion and site of the lesion) may limit the accuracy of LSM by TE, and thus limit the applicability of Baveno criteria in HCC patients. Yet despite that, our study found patients with right-sided tumour involvement still had an acceptable VNT missed rate of 2.5% (five patients) when applying the Baveno criteria. In addition, the difference in VNT missed rate between right and non-right-sided involvement was also insignificant (five patients or 19.2% vs. two patients or 13.3%; *p*-value: 1; Supplementary Table S4). In order to avoid these tumour-related factors that may confound the accuracy of LSM, spleen stiffness measurement may be a useful addition or alternative to improve the non-invasive prediction of VNT in HCC patients. Further studies in this area should be explored.

There are a few limitations to this study. Our study has a relatively small sample size, and a lack of diversity with regard to the underlying aetiology of HCC. The majority of patients had chronic hepatitis B, which remains endemic in our locality. Non-alcoholic fatty liver disease, which has a prevalence of 30% in Hong Kong, is under-represented [26]. These clinical characteristics may infer better applicability of our findings to the Asia-Pacific region than in the West. Nevertheless, Baveno VII criteria have been shown to maintain a high reliability in ruling out VNT in different ethnicities or aetiologies including virus-, alcohol- and non-obese NASH-related cACLD [27]. The use of the ANTICIPATE-NASH model (with LSM, PLT and BMI) in overcoming the low positive predictive value in obese NASH-related cACLD still requires further evaluation [28]. The predominance of viral hepatitis in this cohort may account for the lower event rate, as it is known that the use of antiviral therapy may change the course of patient outcomes and reduce bleeding events.

There was also a relatively low number of patients who received systemic therapy, with the majority being sorafenib due to reimbursement policies and availability in our locality. The lack of diversity in the type of systemic therapy used limited our ability to conduct a comparative analysis amongst various agents, and may not fully reflect the effects of the current first-line standard-of-care regimen of atezolizumab plus bevacizumab, which is thought to have the highest AVB risk amongst the different systemic therapies. Further, 12% of patients defaulted TE measurements and therefore had missing LSM data. This may lead to bias and reduced statistical power. This study also included a heterogenous cohort of patients ranging from BCLC stage A to C, as well as different therapeutic options that may infer different bleeding risk as well as prognosis. Nevertheless, sub-analysis of different alternative therapies revealed an acceptable VNT missed rate throughout all subgroups (Supplementary Table S5). Other limitations include inter-observer variability in defining VNT as the upper endoscopy examinations were conducted by different endoscopists. Lastly, ALT is a well-known confounding factor on LSM, which is often falsely high in the setting of grossly elevated ALT. This effect should be minimal in our cohort as most of our patients had been receiving antiviral treatment and none of the patients had baseline ALT more than five times the upper limit of normal [29].

Further validation with a larger sample size including more patients on systemic therapy, especially those on combination atezolizumab and bevacizumab, is urgently needed. Sub-analysis of VNT risk for different types of systemic therapies would also be informative. To ensure the accuracy of Baveno VII criteria throughout the patient journey of HCC treatment, further studies should be conducted on evaluating the optimal interval for the monitoring of LSM and PLT levels in advanced HCC patients. With a more rapid deterioration expected, more frequent surveillance with Baveno criteria may be beneficial in this particular group of patients, yet the optimal interval for surveillance remains to be examined. Optimal non-invasive prophylactic strategies, such as the plausibility of NSBB in HCC patients, should be further investigated, as current prospective evidence is lacking [30]. Whether empirical use of NSBB can negate the need for endoscopy by empirically reducing AVB risk is also not yet known.

## 4. Conclusions

In this study of predominantly HBV-related advanced HCC, the Baveno VII criteria was were shown to be accurate in identifying varices needing treatment and patients at high risk of developing hepatic events. The Baveno VII criteria can be used cautiously in HCC patients who require systemic therapy, as well as those with right-sided disease. Future prospective studies on how to further broaden the applicability of Baveno VII criteria to other subgroups of HCC aetiologies, identifying optimal cut-offs and incorporating patients treated with combination systemic therapies. are urgently needed.

## Figures and Tables

**Table 1:** Characteristics of included patients with advanced HCC.

	All Patients N = 200	Systemic Therapies N = 76	No Systemic Therapies N = 124	*p*-Value
Follow-up duration (months)	11.9 (4.6–39.6)	8.5 (4.7–21.3)	15.6 (4.5–42.2)	0.066
Male gender	161 (80.5%)	64 (84.2%)	97 (78.2%)	0.3
Age (year)	61.3 (11.3)	58.0 (10.4)	63.4 (11.3)	<0.001
Chronic hepatitis B	139 (69.5%)	54 (71.1%)	85 (68.5%)	0.709
Chronic hepatitis C	15 (7.5%)	7 (9.2%)	8 (6.5%)	0.472
Platelet (×10^9^/L)	204.1 (113.6)	214.2 (117.9)	197.9 (110.8)	0.328
≥150 × 10^9^/L	125 (62.5%)	53 (69.7%)	72 (58.1%)	0.098
≥110 × 10^9^/L	159 (79.5%)	64 (84.2%)	95 (76.6%)	0.196
Prothrombin time (s)	11.9 (1.6)	11.9 (1.0)	12.0 (1.9)	0.738
Creatinine (μmol/L)	82.9 (31.2)	80.8 (38.4)	84.2 (26.0)	0.455
Albumin (g/l)	37.0 (5.3)	37.3 (5.1)	36.9 (5.4)	0.574
Total bilirubin (μmol/L)	20.4 (16.2)	19.3 (13.2)	20.9 (17.8)	0.626
ALT (IU/L)				
Median (IQR)	45 (32–67)	42 (33.5–52.5)	46.5 (30.5–75)	0.160
AFP (μg/L)				
Median (IQR)	213 (11–7757)	1526 (22–44,248)	56 (7.5–2085)	0.005
CTP grade				
A	135 (67.5%)	54 (71.1%)	81 (65.3%)	0.401
B	64 (32.0%)	22 (28.9%)	42 (33.9%)	0.469
C	1 (0.5%)	1 (0.8%)	0 (0%)	1.000
CTP score	6 (5–7)	6 (5–7)	6 (5–7)	0.272
BCLC				
A	25 (12.5%)	7 (9.2%)	18 (14.5%)	0.271
Fulfil Baveno criteria (n = 41)	3 (7.3%)	1 (7.7%)	2 (7.1%)	1.000
Not fulfil Baveno criteria (n = 159)	22 (13.8%)	6 (9.5%)	16 (16.7%)	0.202
B	51 (25.5%)	19 (25%)	32 (25.8%)	0.899
Fulfil Baveno criteria (n = 41)	14 (34.1%)	3 (23.1%)	11 (39.3%)	0.481
Not fulfil Baveno criteria (n = 159)	37 (23.3%)	16 (25.4%)	21 (21.9%)	0.607
C	124 (62%)	50 (65.8%)	74 (59.7%)	0.387
Fulfil Baveno criteria (n = 41)	24 (58.5%)	9 (69.2%)	15 (53.6%)	0.344
Not fulfil Baveno criteria (n = 159)	100 (62.9%)	41 (65.1%)	59 (61.5%)	0.644
Combination with immunotherapy	3 (1.5%)	3 (3.9%)	0 (0%)	0.054
LSM (kPa) Missing (%)	25.4 (11.9–66.8) 12%	32.4 (16.5–69.1) 14.5%	21.8 (11.1–65.2) 10.5%	0.117
IQR/LSM (%) Missing (%)	15.2 (7.3–22.6) 21.2%	14.2 (7.7–22.8) 23.7%	15.5 (7.2–21.7) 20.2%	0.850
With right-sided involvement (n = 91)	17.2 (10–46.7)	19.4 (9.0–24.7)	16.5 (10.0–56.9)	0.457
IQR/LSM (%) Missing (%)	16.4 (9.4–22.4) 24.2%	16.9(13.9–22.9) 34.6%	16.4 (8.0–21.3) 20%	0.419
Without right-sided involvement (n = 109)	35.8 (14.1–75)	48.4 (24.8–75)	24.6 (13.5–75)	0.087
IQR/LSM (%) Missing (%)	14.0 (5.4–22.6) 19.3%	13.5 (0–22.7) 18%	14.4 (6.4–21.7) 20.3	0.641
≤15 kPa	60 (30%)	16 (21.1%)	44 (35.5%)	0.042
≤20 kPa	73 (36.5%)	21 (27.6%)	52 (41.9%)	0.059
≤25 kPa	88 (44%)	27 (35.5%)	61 (49.2%)	0.086
LSM and platelet criteria				
LSM ≤ 15 kPa and PLT > 150 × 10^9^/L	37 (18.5%)	12 (15.8%)	25 (20.2%)	0.440
LSM ≤ 15 kPa and PLT > 110 × 10^9^/L	47 (23.5%)	14 (18.2%)	34 (27.0%)	0.185
LSM ≤ 20 kPa and PLT > 150 × 10^9^/L	41 (20.5%)	13 (17.1%)	28 (22.6%)	0.352
LSM ≤ 20 kPa and PLT > 110 × 10^9^/L	54 (27%)	15 (19.7%)	39 (31.5%)	0.07
LSM ≤ 25 kPa and PLT > 150 × 10^9^/L	53 (26.5%)	17 (22.4%)	36 (29.0%)	0.3
LSM ≤ 25 kPa and PLT > 110 × 10^9^/L	67 (33.5%)	20 (26.3%)	47 (37.9%)	0.092

The data are shown in number (percentage) and mean (standard deviation); data presented in median (interquartile range) are specified. ALT = alanine aminotransferase; AFP = alpha-fetoprotein; CTP = Child–Turcotte–Pugh; HBeAg = hepatitis B e antigen; HBsAg = hepatitis B surface antigen; HBV = hepatitis B virus; HCV = hepatitis C virus; IQR = interquartile range; LSM = liver stiffness measurement; N.A.= not applicable; PLT = platelet.

**Table 2:** Clinical details of detected varices.

	All Patients N = 200	Systemic Therapies N = 76	No Systemic Therapies N = 124	*p*-Value
Any varices	50 (25%)	18 (23.7%)	32 (25.8%)	0.737
Oesophageal varices	48 (24%)	18 (23.7%)	30 (24.2%)	0.935
Gastric varices	4 (2%)	2 (2.6%)	2 (1.6%)	0.626
Acute variceal bleeding (AVB)	11 (5.5%)	3 (3.9%)	8 (6.5%)	0.353
Bleeding oesophageal varices	8 (3.5%)	1 (1.3%)	7 (5.6%)	0.138
Bleeding gastric varices	3 (1.5%)	2 (2.6%)	1 (0.8%)	0.559
Endoscopic therapy	22 (11%)	9 (11.8%)	13 (10.5%)	0.766
Varices needing treatment (VNT)	45 (22.5%)	15 (19.7%)	30 (24.2%)	0.464
Fulfil Baveno criteria (n = 41)	7 (17.1%)	2 (15.4%)	5 (15.4%)	1.000
Not fulfil Baveno criteria (n = 159)	38 (23.9%)	13 (20.6%)	25 (26.0%)	0.434
CTP grade for VNT				
A	24 (12%)	10 (13.2%)	14 (11.3%)	0.693
B	21 (10.5%)	5 (6.6%)	16 (12.9%)	0.157
BCLC				
A	8 (4%)	2 (2.6%)	6 (4.8%)	0.712
B	16 (8%)	6 (7.9%)	10 (8.1%)	0.966
C	21 (10.5%)	7 (9.2%)	14 (11.3%)	0.641
Portal vein thrombosis (PVT) **	75 (37.5%)	36 (47.4%)	39 (31.5%)	0.024
VNT in those with PVT (n = 75)	12 (16.0%)	7 (19.4%)	5 (12.8%)	0.434
Bleeding risk	4 (5.3%)	1 (2.8%)	3 (7.7%)	0.616
Without PVT	125 (62.5%)	40 (52.6%)	85 (68.5%)	0.024
VNT in those without PVT (n = 125)	33 (26.4%)	8 (20%)	25 (29.4%)	0.265
Bleeding risk	6 (4.8%)	2 (5%)	4 (4.7%)	1.000
VNT in following subgroups				
LSM ≤ 15 kPa and PLT ≥ 150 × 10^9^/L	6 (3%)	2 (2.6%)	4 (3.2%)	1.000
LSM ≤ 15 kPa and PLT ≥ 110 × 10^9^/L	9 (4.5%)	3 (3.9%)	6 (4.8%)	1.000
LSM ≤ 20 kPa and PLT ≥ 150 × 10^9^/L	7 (3.5%)	2 (2.6%)	5 (4.0%)	1.000
LSM ≤ 20 kPa and PLT ≥ 110 × 10^9^/L	10 (5%)	3 (3.9%)	7 (5.6%)	0.745
LSM ≤ 25 kPa and PLT ≥ 150 × 10^9^/L	7 (3.5%)	2 (2.6%)	5 (4.0%)	0.711
LSM ≤ 25 kPa and PLT ≥ 110 × 10^9^/L	11 (5.5%)	4 (5.3%)	7 (5.6%)	1.000
Time to VNT (months)	11.3 (3.9–37.4)	7.9 (4.2–21.1)	15.4 (3.8–41.8)	0.067

The data are shown in number (percentage). LSM = liver stiffness measurement; PLT = platelet. ** The *p*-value of VNT rates in patients with vs. without PVT is 0.088.

**Table 3:** Hepatic events of advanced HCC patients.

	All Patients N = 200	Systemic Therapies N = 76	No Systemic Therapies N = 124	*p*-Value
Any hepatic events *	56 (28%)	18 (23.7%)	38 (30.6%)	0.287
Acute variceal bleed (AVB) ^	11 (5.5%)	3 (3.9%)	8 (6.5%)	0.353
Ascites ^	44 (22%)	13 (17.1%)	31 (25%)	0.191
Spontaneous bacterial peritonitis (SBP) ^	4 (2%)	0 (0%)	4 (3.2%)	0.300
Hepatic encephalopathy (HE) ^	17 (8.5%)	5 (6.6%)	12 (9.7%)	0.446
Hepatorenal syndrome (HRS) ^	6 (3%)	1 (1.3%)	5 (4.0%)	0.411
Liver-related death	128 (64%)	54 (71.1%)	74 (59.7%)	0.104
Fulfil Baveno criteria (n = 41)	16 (39.0%)	7 (53.8%)	9 (32.1%)	0.185
Not fulfil Baveno criteria (n = 159)	112 (70.4%)	47 (74.6%)	65 (67.7%)	0.351
Hepatic events in following subgroups				
LSM ≤ 15 kPa and PLT ≥ 150 × 10^9^/L	4 (2%)	1 (1.3%)	3 (2.4%)	1.000
LSM ≤ 15 kPa and PLT ≥ 110 × 10^9^/L	8 (4%)	1 (1.3%)	7 (5.6%)	0.159
LSM ≤ 20 kPa and PLT ≥ 150 × 10^9^/L	6 (3%)	1 (1.3%)	5 (4.0%)	0.411
LSM ≤ 20 kPa and PLT ≥ 110 × 10^9^/L	10 (5%)	1 (1.3%)	9 (7.3%)	0.093
LSM ≤ 25 kPa and PLT ≥ 150 × 10^9^/L	7 (3.5%)	2 (2.6%)	5 (4.0%)	0.711
LSM ≤ 25 kPa and PLT ≥ 110 × 10^9^/L	12 (6%)	3 (3.9%)	9 (7.3%)	0.541
LSM > 25 kPa	28 (14%)	10 (13.2%)	18 (14.5%)	0.786
Fulfil Baveno criteria (n =41)	6 (14.6%)	1 (7.7%)	5 (17.9%)	0.645
Not fulfil Baveno criteria (n = 159)	50 (31.4%)	17 (27.0%)	33 (34.4%)	0.326

The data are shown in number (percentage). * Not including liver-related deaths. ^ One patient might have more than one kind of hepatic event. LSM = liver stiffness measurement; PLT = platelet.

**Table 4:** Predictors of VNT (univariate and multivariate analysis).

Parameters	Univariate Analysis		Multivariate Analysis	
	SHR (95% CI)	*p*-Value	Adjusted SHR (95% CI)	*p*-Value
Use of systemic therapy	0.81 (0.44–1.49)	0.508	1.01 (0.41–2.45)	0.989
Age	0.99 (0.97–1.01)	0.373	0.97 (0.94–1.01)	0.125
Male gender	1.76 (0.79–3.95)	0.168	1.58 (0.79–3.18)	0.198
- BCLC				
- A	Referent		Referent	
- B	0.97 (0.44–2.14)	0.945	1.17 (0.58–2.38)	0.663
- C	0.48 (0.22–1.03)	0.059	0.87 (0.39–1.90)	0.718
- Child–Pugh staging				
- A	Referent		Referent	
- B or C	0.63 (0.30–1.32)	0.224	0.83 (0.38–1.83)	0.646
Presence of PVT	0.55 (0.29–1.03)	0.064	0.77 (0.34–1.77)	0.542
Previous hepatectomy or TACE	1.35 (0.62–2.95)	0.451	0.70 (0.21–2.30)	0.555
LSM	0.99 (0.98–1.00)	0.128	1.00 (0.98–1.01)	0.852
Platelet	0.99 (0.99–0.99)	<0.001	0.99 (0.98–1.00)	0.001
Baveno criteria	0.72 (0.33–1.59)	0.418	1.14 (0.33–3.97)	0.842

SHR = subdistribution hazard ratio; CI = confidence interval.

**Table 5:** Predictors of hepatic events (univariate and multivariate analysis).

Parameters	Univariate Analysis		Multivariate Analysis	
	SHR (95% CI)	*p*-Value	Adjusted SHR (95% CI)	*p*-Value
Use of systemic therapy	0.76 (0.44–1.33)	0.333	1.13 (0.55–2.34)	0.731
Age	1.02 (0.99–1.04)	0.2	1.01 (0.97–1.04)	0.672
Male gender	1.00 (0.52–1.90)	0.992	1.21 (0.62–2.38)	0.57
- BCLC				
- A	Referent		Referent	
- B	0.90 (0.39–2.11)	0.815	1.14 (0.48–2.71)	0.769
- C	0.78 (0.37–1.67)	0.525	1.13 (0.47–2.68)	0.785
- Child–Pugh staging				
- A	Referent		Referent	
- B or C	1.46 (0.83–2.55)	0.187	1.48 (0.75–2.91)	0.261
Presence of PVT	0.81 (0.47–1.39)	0.448	0.83 (0.42–1.64)	0.589
previous hepatectomy or TACE	0.61 (0.26–1.45)	0.264	0.48 (0.15–1.48)	0.199
LSM	1.00 (0.99–1.01)	0.668	1.01 (0.99–1.02)	0.349
Platelet	0.99 (0.99–1.00)	0.004	0.99 (0.99–1.00)	0.005
Baveno criteria	0.41 (0.18–0.94)	0.036	0.78 (0.26–2.33)	0.65

SHR = subdistribution hazard ratio; CI = confidence interval.

## Data Availability

The data presented in this study are available in this article and the supplementary material.

## Supplementary Materials

The following supporting information can be downloaded at: https://www.mdpi.com/article/10.3390/cancers15092480/s1, Figure S1: VNT according to BCLC stage; Figure S2: VNT according to whether patients received systemic therapy; Figure S3: Bleeding events according to whether patients received systemic therapy; Table S1: Any HCC treatment before receiving systemic therapy; Table S2: Alternative therapy given in subgroup of patients who did not receive systemic therapy; Table S3: Sub-analysis according to presence of PVT; Table S4: Sub-analysis according to right or non-right sided tumour involvement; Table S5: Sub-analysis according to different alternative treatments.
