# Supplementary material for: Baveno VII Criteria Is an Accurate Risk Stratification Tool to Predict High-Risk Varices Requiring Intervention and Hepatic Events in Patients with Advanced Hepatocellular Carcinoma
Source: Cancers (Basel). 2023 Apr 26;15(9):2480. doi: 10.3390/cancers15092480 (PMC10177352; doi:10.3390/cancers15092480)
Supplement: Supplementary file 1 [file cancers-15-02480-s001.zip › cancers-2219139-supplementary.pdf]

Supplementary Figures

Supplementary Figure S1: VNT according to BCLC stage

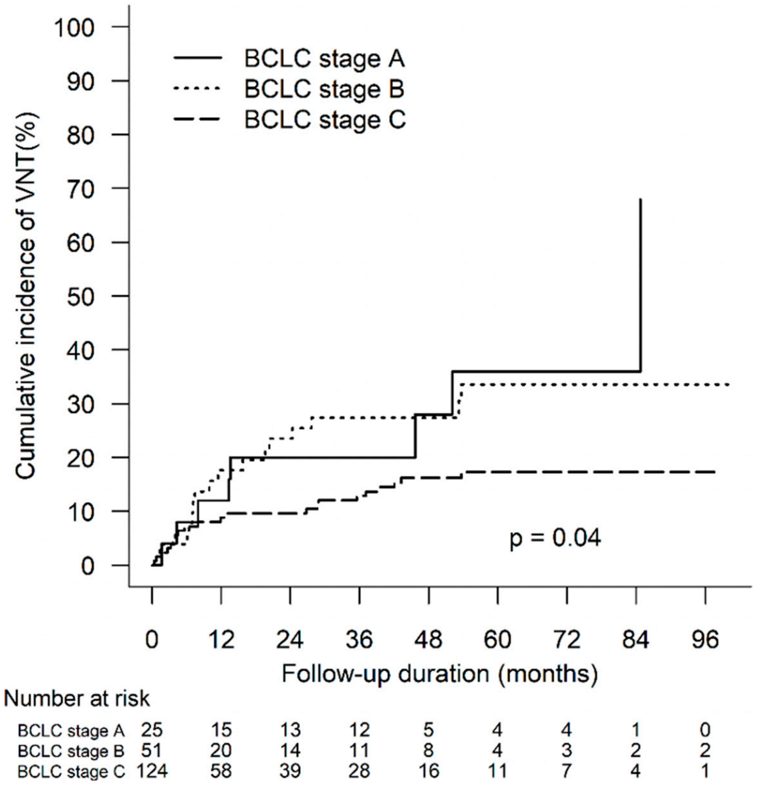

Supplementary Figure S2: VNT according to whether patients received systemic therapy

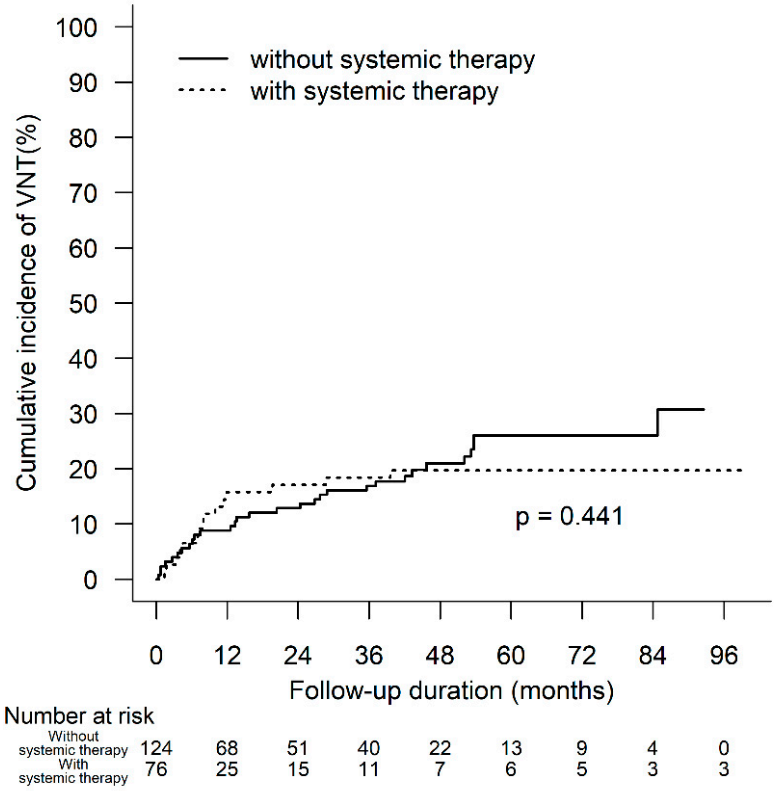

Supplementary Figure S3: Bleeding events according to whether patients received systemic therapy

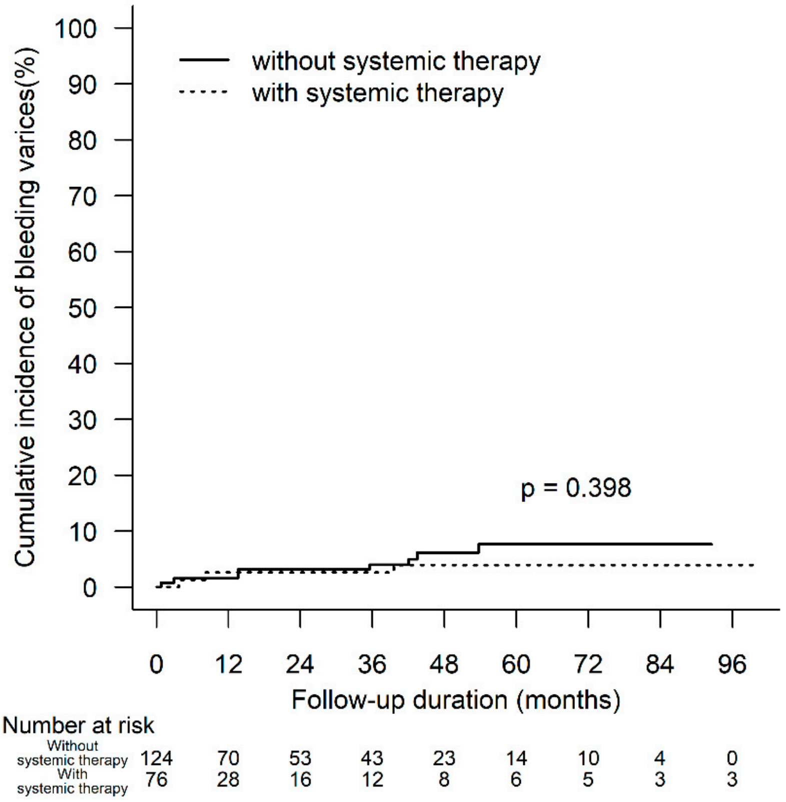

Supplementary Tables

Supplementary Table S1: Any HCC treatment before receiving systemic therapy

Treatment received before systemic therapies (n=76)	27 (35.5%)
Partial hepatectomy (not involving right side)	4 (14.8%)
Partial hepatectomy (Right side)	4 (14.8%)
TACE	17 (63.0%)
RF ablation	2 (7.4%)

Supplementary Table S2: Alternative therapy given in subgroup of patients who did not receive systemic therapy

**Alternative therapy if systemic therapies not given: (n=124) ^**	75 (60.5%)
Partial hepatectomy	20 (16.1%)
TACE	49 (39.5%)
RF ablation	6 (4.8%)
**No treatment/ palliative care**	49 (39.5%)

Supplementary Table S3: Sub- analysis according to presence of PVT

	**All patients**	**With PVT (n=75)**	**No-PVT (n = 125)**	**P-value**
**Fulfil Baveno criteria**	41 (20.5%)	11 (14.7%)	30 (24%)	0.113
**VNT missed rate (n=41)**	7 (3.5%)	2 (1%)	5 (2.5%)	1.000

Supplementary Table S4: Sub-analysis according to right or non-right sided tumour involvement

	**All patients**	**Right sided involvement (n = 91)**	**Non-right sided involvement (n = 109)**	**P-value**
**VNT count**	45 (22.5%)	23 (25.3%)	22 (20.2%)	0.391
**Fulfil Baveno criteria (n=41)**	7 (17.1%)	5 (19.2%)	2 (13.3%)	1.000
**Not fulfil Baveno criteria (n=159)**	38 (23.9%)	18 (27.7%)	20 (21.3%)	0.351

Supplementary Table S5: Sub-analysis according to different alternative treatments

	**Partial hepatectomy**	**TACE**	**RF ablation**	**No treatment**
**Fulfil Baveno criteria (n =41)**	10	12	2	4
**VNT:**	1 (0.5%)	3 (1.5%)	0 (0%)	0 (0%)
**Not fulfil Baveno criteria (n = 159)**	10	37	4	45
**VNT:**	2 (1%)	14 (7%)	2 (1%)	7 (3.5%)
